# Fabrication of low-density GaN/AlN quantum dots *via* GaN thermal decomposition in MOCVD

**DOI:** 10.1186/1556-276X-9-341

**Published:** 2014-07-09

**Authors:** Jin Zhang, Senlin Li, Hui Xiong, Wu Tian, Yang Li, Yanyan Fang, Zhihao Wu, Jiangnan Dai, Jintong Xu, Xiangyang Li, Changqing Chen

**Affiliations:** 1 Wuhan National Laboratory for Optoelectronics, School of Optical and Electronic Information, Huazhong University of Science and Technology, Wuhan 430074, People’s Republic of China; 2 Key Laboratory of Infrared Imaging Materials and Detectors, Shanghai Institute of Technical Physics, Chinese Academy of Sciences, Shanghai 200083, People’s Republic of China

**Keywords:** GaN quantum dots, Thermal decomposition, Low density, MOCVD

## Abstract

With an appropriate high anneal temperature under H_2_ atmosphere, GaN quantum dots (QDs) have been fabricated *via* GaN thermal decomposition in metal organic chemical vapor deposition (MOCVD). Based on the characterization of atomic force microscopy (AFM), the obtained GaN QDs show good size distribution and have a low density of 2.4 × 10^8^ cm^-2^. X-ray photoelectron spectroscopy (XPS) analysis demonstrates that the GaN QDs were formed without Ga droplets by thermal decomposition of GaN.

## Background

Low-dimensional III-nitrides materials have gained much research attention because of their strong carrier confinement which may lead to the realization of next-generation electronic and optoelectronic applications [1-5]. Among these low-dimensional III-nitride materials, the study of single GaN quantum dot has become the recent focus due to its promising applications in the solid-state quantum computation, single-photon sources, and single-photon detectors, in which the density of quantum dots is required to be as low as approximately 10^8^ cm^-2^[6-9]. However, challenges remains in fabrication of low-density GaN quantum dots (QDs) with high quality. On the one hand, the most frequently used fabrication approach is self-assembly process *via* Stranski-Krastanov (SK) growth mode which requires sufficient lattice mismatch, but it is harder to acquire low-density GaN QDs and usually results in randomly distributed QDs with different sizes [10,11]. On the other hand, although some low-density GaN nanodots can be obtained by the droplet epitaxy technique based on a vapor-liquid-solid process which offers distinct advantages in size and density manipulation of QDs, the droplet epitaxy technique usually results in QDs with the incomplete transition from Ga droplet to crystal GaN. What is more, there is almost no report about fabrication of low-density GaN QDs *via* the droplet epitaxy technique [12,13]. Motivated by the above issues, recently, we have demonstrated the fabrication of GaN nanodots on AlN templates *via* GaN thermal decomposition in H_2_ atmosphere, which does not involve the induction of strain or the crystallization of the Ga droplets [14]. In addition, the recent studies and applications of GaN-based materials growth have been demonstrated [15-20]. In this letter, the thermal decomposition conditions are further optimized and low-density GaN/AlN QDs with high quality are achieved. This study provides an alternative approach to fabricating low-density GaN QDs for single-photon devices.

## Methods

**Figure 1:**
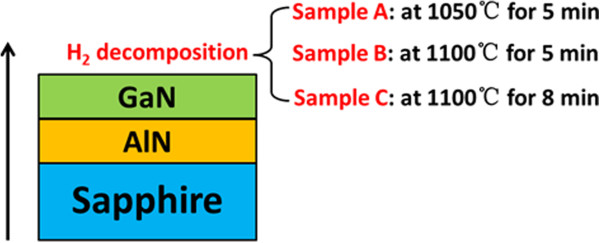
**The schematic of H**_**2**_-**annealed conditions of samples A, B, and C.**

GaN QDs were formed on AlN/sapphire templates by metal organic chemical vapor deposition (MOCVD). Triethylgallium (TEGa), trimethylaluminum (TMAl), and ammonia were used as precursors for Ga, Al, and N sources with H_2_ as carrier gas. The total pressure was maintained at 40 Torr. The sapphire substrates were introduced into the MOCVD reactor and 800-nm-thick AlN buffer layers were deposited. Then, 800-nm-thick GaN epilayers were grown on the AlN templates at 940°C. Subsequently, the samples were annealed in H_2_ atmosphere at different conditions: sample A, at 1,050°C for 5 min; sample B, at 1,100°C for 5 min and sample C, at 1,100°C for 8 min, as shown in Figure 1. After that, these samples were cooled down to room temperature at the presence of NH_3_ + H_2_. Besides, two controlled experiments were also conducted. One was the growth of 800-nm-thick GaN on 800-nm-thick AlN/sapphire without decomposition in H_2_ (sample D), and another one is an 800-nm-thick AlN buffer template on sapphire without decomposition in H_2_ (sample E). The surface morphologies of all samples were characterized by atomic force microscopy (AFM) measurements. The surface chemistries of obtained GaN QDs and some control samples were investigated using X-ray photoelectron spectroscopy (XPS) measurements with monochromatic Mg Kα X-ray source (hν = 1,253.6 eV).

## Results and discussion

**Figure 2:**
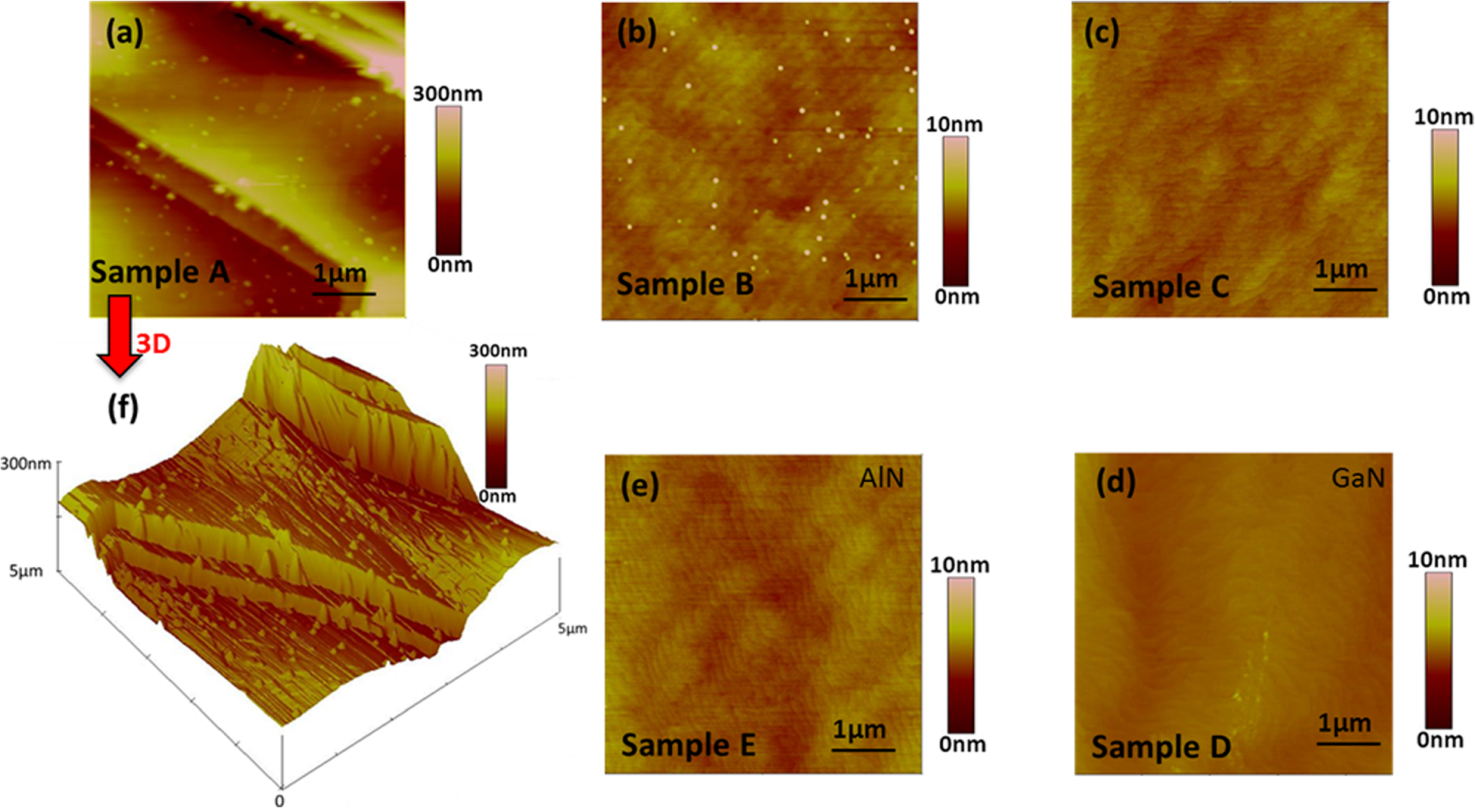
**AFM images of samples.** Samples **(a)** A, **(b)** B, **(c)** C, **(d)** D, **(e)** E, and **(f)** corresponding the 3D AFM image of sample A.

The surface morphologies of all samples were studied by atomic force microscopy (AFM), and the results are shown in Figure 2. Compared with the surface morphology of controlled sample D (Figure 2d), it is obvious that GaN decomposition occurs for Sample A (Figure 1a). Figure 1f is the corresponding three-dimensional (3D) AFM image of Figure 1a, in which distributed dots are on terraces and abrupt peaks are to be buried in the side wall, indicating the decomposition process for the formation of GaN dots. As the decomposition occurred toward the inner of the side wall, the abrupt peaks are then exposed to H_2_ flow and decomposed. Since the heights of peaks decrease faster than the diameters of peaks, the side wall is etched away and the peaks are etched to small dots with a longer etching time, which is consistent with our previous observation [14]. With increasing of the annealing temperature from 1,050°C to 1,100°C, the decomposition of GaN has an interesting phenomenon that the steps disappear and well-shaped dots are just left on a flat surface, as shown in Figure 2b. The obtained GaN QDs show a low density in the magnitude of approximately 10^8^ cm^-2^. As expected, these dots are etched as the elongation of annealing time from 5 to 8 min, left with atomically flat surface (Figure 2c) similar to that of controlled sample E (Figure 2e). It is clear that surface morphology of the AlN buffer templates before and after annealing in H_2_ are exactly the same, indicating that no decomposion of AlN takes place at the temperature of 1,100°C. This result is in good agreement with the claim made by Y. Kumagai et al. [21].

**Figure 3:**
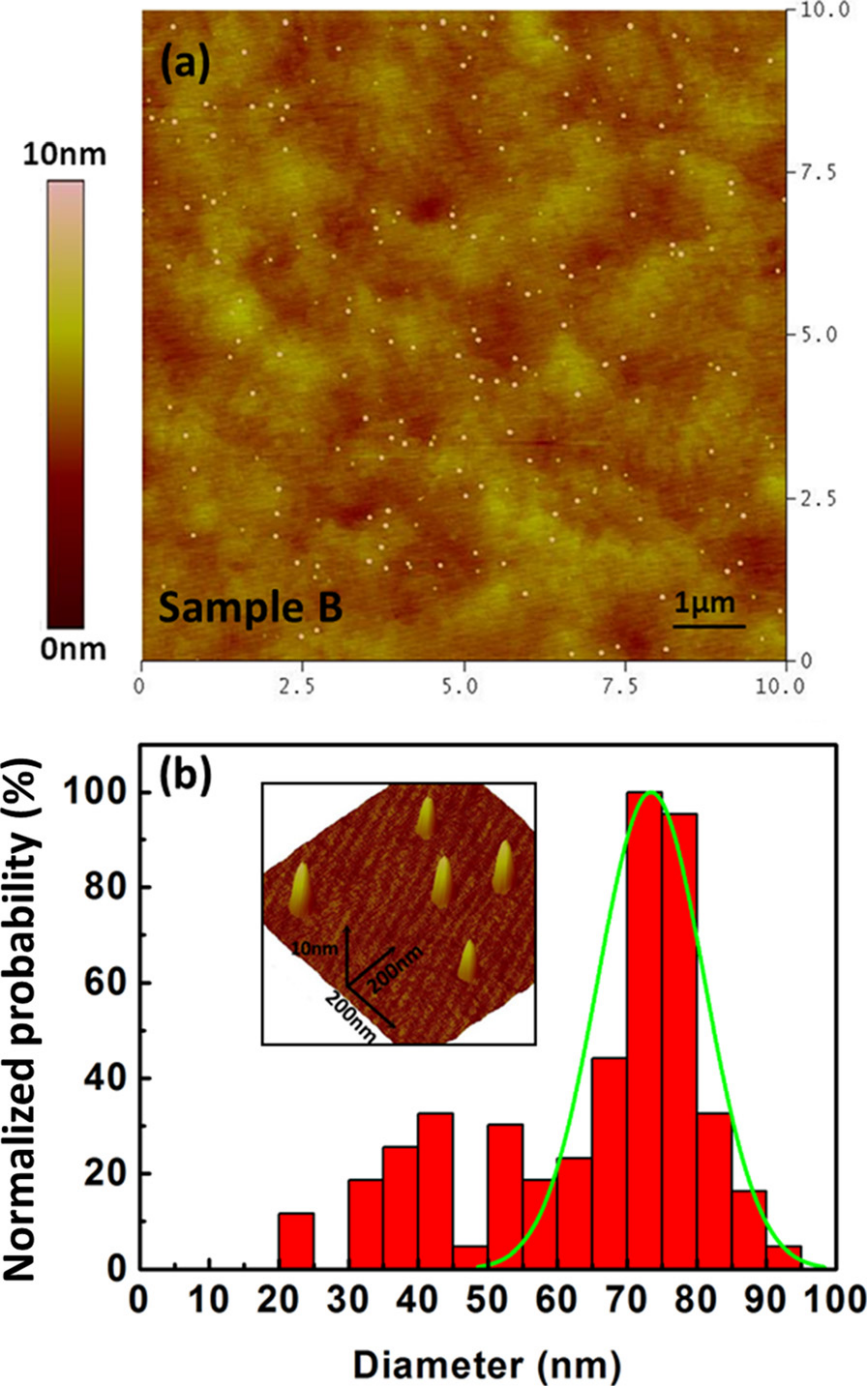
**AFM images of sample B (a) and diameter distributions of GaN QDs (b). (a)** Scan area 10 × 10 μm^2^; **(b)** Analyzed from the AFM images of sample B. Inset is the 3D image of obtained GaN QDs.

To further investigate the size distribution of the obtained GaN QDs, the AFM images of sample B with scan area 10 × 10 μm^2^ is shown in Figure 3a. The QDs have a low density of approximately 2.4 × 10^8^ cm^-2^ and no obvious big dots are observed, showing the good uniformity. Figure 3b illustrates the diameter histograms obtained by the AFM analysis of Figure 3a. It is clear that the most probable diameter is in the range from 70 to 80 nm. The inset of Figure 3b shows a detailed 3D AFM image of the QDs in 1 × 1 μm^2^, indicating the similar well-formed dot structure. According to the results above, the obtained GaN QDs have a good size distribution. To the best of our knowledge, this is the first report of low-density GaN QDs fabricated *via* GaN thermal decomposition in MOCVD.

**Figure 4:**
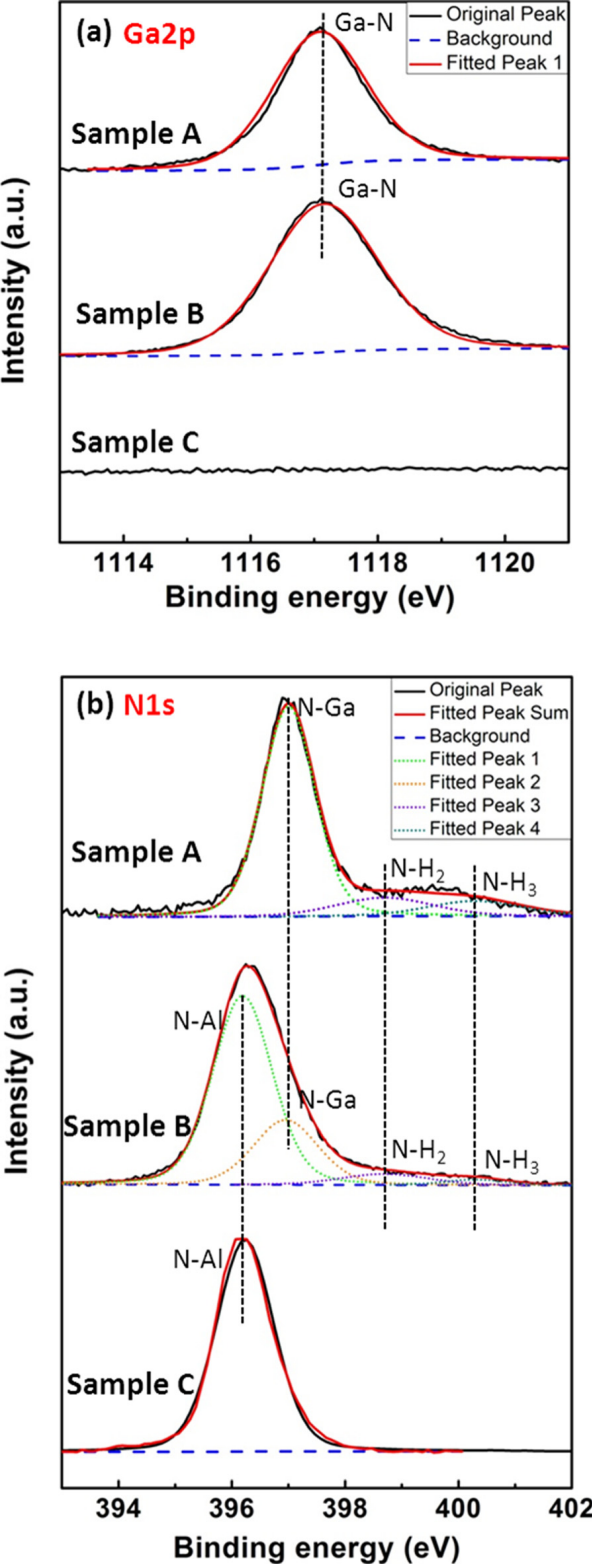
**XPS spectra of (a) Ga2*p *and (b) N1*s *for samples A, B, and C.** The background lines and the fitted lines were also subtracted.

As is shown in Figure 4, since XPS analysis was performed for samples A, B, and C, Ga2*p* and N1*s* core level spectra were measured. For both of the XPS spectra, the C1*s* peak at approximately 285.0 eV was used as binding-energy reference. Baselines were fixed using a Shirley background subtraction model and all peaks were fitted using a linear combination of 80% Gaussian and 20% Lorentzian line shapes. On the one hand, the Ga2*p* spectra are analyzed in Figure 4a. Both samples A and B have a Ga2*p* peak which can be fitted as only one subpeak located at 1,117.1 eV, which is assigned to Ga-N bond [22-24]. So there are no Ga droplets but GaN on the surface of samples A and B, indicating that the Ga desorption rate exceed the GaN decomposition rate. On the contrary, if the Ga desorption rate is less than the GaN decomposition rate, Ga droplets will generate in a chemical manner and Ga-Ga bond will be observed. No Ga2*p* peaks were observed in sample C, confirming that sample C is just the AlN buffer after H_2_ decomposition. On the other hand, the N1*s* spectra are analyzed in Figure 4b. For sample A, the N1*s* spectra can be decomposed into a total of four fitted subpeaks at 397.0, 398.7, and 400.3 eV, which were assigned to N-Ga bond, N-H_2_ bond and N-H_3_ bond [25,26], respectively. Only GaN existed on the surface of sample A. For sample C, the N1*s* spectra can be decomposed into one subpeaks at 398.7 eV, which is assigned to N-Al bond [27]. Only AlN existed on the surface of sample C. For sample B, the N1*s* spectra were decomposed into a total of four fitted subpeaks at 396.2, 397.0, 398.7, and 400.3 eV, which can be assigned to N-Al bond, N-Ga bond, N-H_2_ bond, and N-H_3_ bond, respectively. These fitted subpeaks coincide with the fitted subpeaks of samples A and C, providing a chemical evidence for the existence of GaN QDs formed on the AlN buffer. In addition, the N-H_2_ bond and N-H_3_ bond were obtained in samples A and B but did not exist in sample C, indicating that the appearance of N-H_2_ bond and N-H_3_ bond were caused by the interaction of decomposed GaN and hydrogen at high temperature.

## Conclusions

In summary, GaN QDs were fabricated on flat AlN buffer *via* H_2_ decomposition of GaN film at high temperature. The AFM measurements show that the obtained GaN QDs have good size uniformity and a low dot density about 2.4 × 10^8^ cm^-2^. The XPS spectra analysis actually demonstrated that the GaN QDs do not contain Ga droplets. The results provide an alternative approach to fabricate low-density GaN QDs for applications in single-photon devices.

## Abbreviations

AFM: atomic force microscopy; 3D: three-dimensional; MOCVD: metal-organic chemical vapor deposition; QDs: quantum dots; S-K: Stranski-Krastanov; TEGa: triethylgallium; TMAl: trimethylaluminum; XPS: X-ray photoelectron spectroscopy.

## Competing interests

The authors declare that they have no competing interests.

## Authors’ contributions

JZ wrote the manuscript and participated in all the experiments and the data analysis. SLL, HX, WT, YL, ZHW, and JND partially participated in the experiments and the data analysis. JTX and XYL offer supporting in the testing of XPS. YYF and CQC supervised the writing of the manuscript and all the experiments. All authors read and approved the final manuscript.

## Authors’ information

JZ, SLL, WT, and YL are PhD students, HX is the postdoctor, JND, YYF and ZHW hold associate professor positions, and CQC is a professor at the Huazhong University of Science and Technology. XYL and JTX hold the researcher and associate researcher positions at the Shanghai Institute of Technical Physics.
